# Anxiety and Depressive Symptom Trajectories in Adolescence and the Co-Occurring Development of Cognitive Biases: Evidence from the CogBIAS Longitudinal Study

**DOI:** 10.1007/s10802-020-00694-9

**Published:** 2020-09-14

**Authors:** Annabel Songco, Charlotte Booth, Olivia Spiegler, Sam Parsons, Elaine Fox

**Affiliations:** Department of Experimental Psychology, University of Oxford, New Radcliffe House, Radcliffe Observatory Quarter, Oxford, OX26GG UK

**Keywords:** Cognitive Bias, Anxiety, Depression, Adolescence, Longitudinal, Growth mixture modeling (GMM)

## Abstract

The development of negative cognitive biases, together with symptoms of anxiety and depression, has yet to be investigated longitudinally. Using a three-wave design, the present study examined developmental trajectories of anxiety and depressive symptoms and the co-occurrence of cognitive biases, in a large normative sample of adolescents (*N* = 504). Data was drawn from the CogBIAS Longitudinal Study (CogBIAS-L-S), which assessed a wide range of psychological variables, including cognitive biases and self-reported anxiety and depressive symptoms, when adolescents were approximately 13, 14.5, and 16 years of age. The results showed that overall levels of anxiety were low and stable, while levels of depression were low but increased slightly at each wave. Growth mixture modeling identified four distinct developmental classes with regard to anxiety and depressive symptoms. Multiple group analysis further showed that class membership was related to the development of cognitive biases. The majority of the sample (75%) was characterised by ‘Low symptoms’ of anxiety and depression and showed low interpretation and memory biases for negative stimuli at each wave. A second class (11%) displayed ‘Decreasing anxiety symptoms’ and showed decreasing interpretation bias, but increasing memory bias. A third class (8%) displayed ‘Comorbid increasing symptoms’ and showed increasing interpretation and memory biases. While the fourth class (6%) displayed ‘Comorbid decreasing symptoms’ and showed decreasing interpretation and memory biases. This longitudinal study sheds light on healthy and psychopathological emotional development in adolescence and highlights cognitive mechanisms that may be useful targets for prevention and early interventions.

Cognitive theories of anxiety and depression emphasise the importance of cognitive biases as key factors contributing to the onset and maintenance of emotional disorders (Beck et al. 1985; Clark et al. 1999). These biases selectively direct information-processing resources towards negative, relative to positive or benign information, resulting in distorted patterns of thinking. Early work suggested that selective attention towards threat-related stimuli was characteristic of anxiety, while memory biases favouring negative self-referent information were characteristic of depression (Mathews and MacLeod 1994). These cognitive biases, together with biased interpretations of ambiguity, are now considered transdiagnostic mechanisms underlying both anxiety and depression (Crick and Dodge 1994; Muris and Field 2008). There is growing evidence that negative biases in attention, interpretation, and memory are mechanisms associated with anxiety and depression in adolescents (Lau and Waters 2016; Platt et al. 2016). However, much of this research has been cross-sectional and little work has investigated the development of cognitive biases longitudinally, which would provide a deeper understanding of the early risk and protective factors precipitating the onset of anxiety and depression in adolescence.

The CogBIAS hypothesis offers a theoretical model, which integrates cognitive biases and genetic factors as risk and protective mechanisms contributing to psychological functioning and resilience (Fox and Beevers 2016). The model proposes that negative cognitive biases, are toxic information-processing styles that lead to a downward spiral of emotional vulnerability, perpetuating emotional disorders such as anxiety and depression. In contrast, emotional resilience is characterised by an upward spiral of positivity and positive cognitive biases that enhance flourishing and optimal mental health. Adolescence is a critical developmental period where fluctuations in emotional vulnerability and emotional resilience may have a profound impact on life trajectories (Fuhrmann et al. 2015). Thus, longitudinal research on cognitive biases would provide important insights into psychological functioning across adolescence.

## Developmental Trajectories of Anxiety and Depression

Developmental trajectories of anxiety and depression in adolescents have been widely investigated with large-scale longitudinal studies. Growth mixture modeling (GMM) is a useful data-driven approach for discerning distinct developmental classes within large heterogeneous data sets and identifying those most at risk (Grimm et al. 2017; Olino et al. 2010). This approach has mostly been applied to developmental trajectories of depression. A recent meta-analysis of twenty longitudinal studies published between 2002 and 2015, found substantial heterogeneity in the development of depression during adolescence (Shore et al. 2018). Within these studies, between three and eleven distinct developmental trajectories were found, although a random pooled effect estimate identified three distinct groups. The largest group identified (56% of the pooled sample), were characterised by low or no depression throughout adolescence. The second group (26%) were characterised by moderate and stable levels of depression. While the third group (12%) showed high fluctuating levels of depression throughout adolescence. Risk factors included being female, having low socio-economic status, as well as multiple peer or family problems, and poor adjustment outcomes.

Age at onset has also been shown to be an important factor contributing to depression heterogeneity in adolescence. A recent study using data from 7543 adolescents who took part in the Avon longitudinal study, a 1991 UK birth cohort, found three distinct depression trajectories (Rice et al. 2019). The largest group (74%) were characterised by persistently low symptoms. The second largest group (17%) were characterised by late-onset (around 16 years of age) and increasing depression. While the smallest group (9%) showed early-onset (around 12 years of age) and increasing depression. Genome-wide analysis was conducted and polygenic risk scores based on different psychiatric disorders were able to distinguish between the groups. Both the late-onset and early-onset groups, relative to the low symptoms group, were associated with higher polygenic risk for major depressive disorder. Furthermore, the early-onset group was associated with higher polygenic risk for attention deficit hyperactivity disorder and schizophrenia, suggesting that this group may have a wider breadth of genetic psychiatric vulnerability. While this study highlights important biological pathways, cognitive mechanisms that could provide useful intervention targets were not investigated.

The development of anxiety in adolescence is less clearly understood. This is partly due to the large number of subtypes of anxiety (e.g., generalised anxiety, separation anxiety, and social phobia), which tend to show different peak ages of onset (Cummings et al. 2014). Previous longitudinal studies have found an overall trend for decreasing levels of anxiety from childhood to adolescence (Allan et al. 2014; McLaughlin and King 2015; Van Oort et al. 2009). However, this is likely to be symptom-specific, as panic disorder and social anxiety have been shown to increase during adolescence (Hale III et al. 2009). One cohort study conducted in a community sample of 2220 adolescents, found that the developmental course of anxiety symptoms decreased from late childhood to early adolescence, however slightly increased from mid-adolescence (generalised anxiety, separation anxiety, and social phobia) or late adolescence (panic disorder and OCD) onwards depending on the anxiety subtype (Van Oort et al. 2009). There is some consensus that anxiety predominately manifests during childhood and early adolescence, while depression develops in later adolescence and young adulthood (Hankin et al. 1998; Merikangas et al. 2010; Roza et al. 2003). Yet, anxiety and depression are highly overlapping and comorbid across adolescence (Ferdinand et al. 2005). Due to this, some have argued that both anxiety and depression be considered under one general factor reflecting ‘internalising disorders’ (Hankin et al. 2016). However, a longitudinal study of 1313 adolescents found that while anxiety and depression are highly comorbid, they are best described by parallel growth processes (Hale III et al. 2009).

An in-depth review of the literature on anxiety and depression comorbidity in youth led to the ‘Multiple Pathways Model’ (Cummings et al. 2014). These authors concluded that anxiety and depression are separate but meaningfully related constructs, which emerge largely from three distinct pathways. Pathway one refers to youth with a diathesis for anxiety (often separation or social anxiety), which develops into depression comorbidity if anxiety is left untreated. In this pathway, anxiety is likely to be severe and depression mild. Pathway two refers to youth with a shared diathesis for anxiety and depression who experience the disorders simultaneously, often manifesting with symptoms of depression and generalised anxiety. In this pathway, anxiety is likely to be severe and depression moderate. Pathway three refers to youth with a diathesis for depression who develop anxiety comorbidity resulting from depression-related impairment, such as peer victimisation or social isolation. The third pathway is the least common and is likely to represent older adolescents and young adults. Thus, evidence suggests that anxiety and depression often co-occur during childhood and adolescence, which can have a detrimental impact on subsequent development (Kaufman et al. 2001). A better understanding of the mechanisms that contribute to the onset and maintenance of early psychopathology in youth can help identify risk factors to target in interventions.

## Development of Cognitive Biases

While previous research has investigated developmental trajectories of anxiety and depression in adolescence, little work has investigated the longitudinal development of cognitive biases. A recent study investigated associations between overgeneral memory bias, rumination, anxiety, and depression across three waves in a sample of 269 adolescents (Gutenbrunner et al. 2018). Overgeneral memory bias is the tendency to recall general or broad memories rather than specific occasions or events (Williams and Broadbent 1986). For instance, when instructed to recall a happy event, a depressed individual with overgeneral memory bias may respond, “when I am on holidays” instead of recalling a specific event such as “when I visited the Grand Canyon for the day”. The study by Gutenbrunner et al. (2018) found that across the entire sample, overgeneral memory bias was not associated with anxiety or depression in adolescents. This is somewhat surprising given some evidence that overgeneral memories constitute a trait-like marker of depression vulnerability in adults and adolescents (Askelund et al. 2019). However, there was evidence for an association between overgeneral memory and prospective increasing levels of anxiety, in a sub-sample of youth who showed increasing levels of rumination. This highlights the importance of distinguishing between healthy youth and those at elevated risk for psychopathology. In another study of 331 youth, three groups were identified based on the developmental trajectory of social anxiety symptoms (Miers et al. 2013). The highest risk group, characterised by high fluctuating symptoms, showed the highest levels of social interpretation bias at baseline. Together, these studies suggest that interpretation and memory biases may lead to prospective increases in internalising symptoms. However, neither study modelled the development of cognitive biases longitudinally. Therefore, more research investigating the development of cognitive biases in large normative samples is needed (Field and Lester 2010).

## Present Study

The aim of the present study was to investigate the developmental trajectories of anxiety and depressive symptoms in adolescents and the co-occurrence of cognitive biases. Cognitive biases are hypothesised to play a key role in the development and maintenance of anxiety and depression in youth (Crick and Dodge 1994; Lau and Waters 2016; Muris and Field 2008; Platt et al. 2016). To the best of our knowledge, this is the first study to examine the development of cognitive biases longitudinally in relation to anxiety and depression symptom trajectories, in a large normative sample of adolescents. Data was drawn from the CogBIAS Longitudinal Study (CogBIAS-L-S), which assessed a wide range of psychological variables at three time points across early to mid-adolescence (Booth et al. 2017). In the present study, interpretation bias, memory bias, and self-reported anxiety and depression were investigated. In the wider study, attention bias was also measured using a pictorial Dot-probe task (MacLeod et al. 1986). However, the Dot-probe task displayed poor psychometric properties and was excluded from further analysis (see Booth et al. 2019, for further information). Therefore, we only investigated the development of interpretation and memory bias in the current study.

The current study used a person-oriented approach (i.e., GMM) to identify distinct developmental classes of anxiety and depressive symptoms across the three waves. First, we conducted a parallel process latent growth curve model for anxiety and depressive symptoms to investigate the overall levels of symptoms and rate of change over time. Following this, we conducted a parallel process GMM analysis to uncover groups of adolescents with distinct developmental class trajectories of anxiety and depression. Finally, we investigated associations between class membership and cognitive biases in memory, social interpretation, and non-social interpretation bias. We hypothesised that adolescents would display high comorbidity between anxiety and depression, but due to the developmental period studied, we expected to find a slight increase in depressive symptoms across the sample and stable or decreasing levels of anxiety. In line with previous research, we expected to find multiple class trajectories, with the majority of the sample showing a healthy trajectory characterised by consistently low symptoms. Although no previous research has examined the development of cognitive biases in adolescents, we expected that they would match those of anxiety and depression, such that increasing symptoms would correspond with increasing negative biases, while decreasing symptoms would correspond with decreasing negative biases.

## Method

### Participants

Data was drawn from the CogBIAS Longitudinal study (CogBIAS-L-S), a three-wave study assessing psychological development during adolescence (Booth et al. 2017). The normative sample comprised of 504 adolescents from 10 different cohorts in the South of England, UK. Adolescents were first assessed near the beginning of secondary school (between the ages of 12 and 14, depending on the school type) and followed for 4 years, completing re-assessment every 12 to 18 months. For the total sample at wave 1 (W1), mean age was 13.4 (*SD* = 0.07; 55% females; 76% Caucasian), at wave 2 (W2) the mean age was 14.6 (*SD* = 0.06; 56% females; 76% Caucasian), and for wave 3 (W3) the mean age was 15.7 (*SD* = 0.06; 58% females; 75% Caucasian). Overall, there was a low dropout rate observed at W2 (11%, *N* = 450) and W3 (19%, *N* = 411), mainly due to school absences on the day of testing or students leaving the school. Socio-economic status (SES) was evaluated as the average score of parent’s highest level of education (1 = “Secondary school”, 2 = “Vocational/technical school”, 3 = “Some college”, 4 = “Bachelor’s degree”, 5 = “Master’s degree”, 6 = “Doctoral degree”). The median level of parental education was 4 (Interquartile range = 2). Please refer to Booth et al. (2019) for a more detailed description of the cohort profile.

### Procedure

Testing sessions were conducted in schools during lessons or at the University of Oxford. Testing was completed in computer labs in groups, ranging in size from 6 to 50 students, depending on the size of the cohort and the available testing space. During the two-hour testing session, participants completed cognitive tasks and self-report questionnaires individually, with a short break after one hour. Participants were instructed to work under exam conditions throughout the session, which meant not talking or looking at their peers’ computer screens. There was at least two researchers present during the testing sessions as well as teachers from the school to help supervise the session. Participation in the study was voluntary and adolescents were compensated by means of a £10 Amazon voucher at the end of each session. Parents provided written informed consent for their child to participate in the study by completing a paper or online version of the Parent Consent Form, depending on the school’s preference. Parents were instructed to read the information sheet and return the completed consent form to the research team, either in paper format or for the online version by selecting a checkbox to consent to their child participating in the study. In addition, on the day of testing, written informed assent was obtained from participants after the study procedure was explained to them. Ethical approval was obtained from the National Health Service (NHS) National Research Ethics Service (NRES) Committee South Central (Project ID: 141833; 14/SC/0128). For a detailed description of the study design see Booth et al. (2017, 2019).

### Measures

#### Anxiety and Depression

The Revised Children’s Anxiety and Depression Scale - Short Form (RCADS-SF; Ebesutani et al. 2012) is a 25-item self-report questionnaire used to assess internalising symptoms. Depression is assessed with 10 items (e.g., “I feel sad or empty”) and Anxiety with 15 items (e.g., “I worry that something bad will happen to me”). The items are scored on a 4-point Likert scale ranging from 0 (“Never”) to 3 (“Always”). A score for Depression and Anxiety was calculated by summing the relevant items. Higher scores indicated greater internalising symptoms. The RCADS-SF is derived from the original 47-item questionnaire (Chorpita et al. 2000) and has shown to have good reliability and validity in children and adolescents (Chorpita et al. 2005). In the current study, internal consistency was high at each wave for Anxiety (Cronbach’s *α* = 0.87, 0.87, 0.87) and Depression (Cronbach’s *α* = 0.86, 0.88, 0.89).

#### Interpretation Bias

The Adolescent Interpretation and Belief Questionnaire (AIBQ; Miers et al. 2008) was used to assess interpretation bias. Participants were presented with 10 ambiguous scenarios. For each scenario, they were asked to indicate how likely each of the three possible interpretations (positive, negative, or neutral) would pop into their mind, using a 5-point Likert scale (1 = “Does not pop in my mind”, 3 = “Might pop in my mind”, 5 = “Definitely pops in my mind”). There were five social scenarios (e.g., “You’ve invited a group of classmates to your birthday party, but a few have not yet said if they are coming”) and five non-social scenarios (e.g., “You’ve received bad marks for your last few tests. Why has this happened?”). A score for each of the subscales (‘Negative Social’, ‘Positive Social’, ‘Negative Non-Social’ and ‘Positive Non-Social’) was calculated as the mean likelihood ratings of the respective items. Bias indices were then computed to create a ‘Social Interpretation bias’ score; (Negative Social – Positive Social) and a ‘Non-social Interpretation bias’ score; (Negative Non-social – Positive Non-social). Higher scores indicated greater negative interpretations for social and non-social situations, respectively. The differential stability was high across waves for the bias indices of ‘Social Interpretation bias’ (ICC_3,1_ = .77) and ‘Non-social Interpretation bias’ (ICC_3,1_ = 0.74).

#### Memory Bias

The Self-Referential Encoding Task was used to assess memory bias. The task was comprised of three phases; In the encoding phase, self-referent adjectives were displayed on the screen for 200 ms, followed by the caption “Describes me?”, after which participants responded ‘yes’ or ‘no’ using the “Y” and “N” keys on the computer keyboard. The word list comprised of 22 positive (e.g., “attractive”) and 22 negative (e.g., “unhappy”) self-referent adjectives that had been matched on word length and recognisability, as well as validated in a previous adolescent sample (Hammen and Zupan 1984). In the distraction phase, participants were prompted to solve three simple mathematics questions displayed on the screen. Finally, in the surprise recall phase, participants were given three minutes to recall and type as many words as they could remember from the “Describes me” task. A memory bias score was calculated as; ((Negative words endorsed and recalled – Positive words endorsed and recalled) / Total number of words endorsed and recalled)). A score of ‘0’ indicated no bias, while lower scores indicated a positive bias, and higher scores indicated a negative bias. The score was computed in this way so that high numbers reflected increased risk for psychopathology. Internal consistency could not be assessed for this count based bias index, but differential stability was high across waves (ICC_3,1_ = 0.72).

### Data Analysis

The current study used a person-oriented approach (i.e., GMM) to identify distinct developmental classes of anxiety and depressive symptom trajectories across the CogBIAS-L-S sample at three waves. To examine the average development of anxiety and depression, we first conducted a parallel process latent growth curve model for anxiety and depressive symptoms. This model estimated intercepts and slopes, which can be interpreted as an adolescent’s initial level of anxiety and depression and rate of change over time. Technically, this was achieved by fixing the time scores of the slope factors to 0 for the W1 manifest variables, and to 1 for the W3 manifest variables. The factor loadings of the W2 manifest variables were freely estimated. As a result, the slope estimates referred to a change between the first and third wave. To improve model fit, we allowed the error variances of the W2 manifest variables to correlate. The slopes were correlated and regressed on the intercepts of the other process (i.e., slope depression on intercept anxiety, slope anxiety on intercept depression).

Second, we identified distinct developmental classes with regard to anxiety and depressive symptoms. We used growth mixture modeling, which allows intercepts and slopes to differ across a set of classes. The variance and covariance of the growth parameters were freely estimated and held equal across classes. This means that individuals within a class can vary around the class-specific intercept and slopes, but across classes the variation was equal. To identify the appropriate number of classes, we first specified an unconditional, parallel process growth mixture model that included two classes. Using a stepwise procedure, we added one additional class k at a time to the model and compared whether the more parsimonious model fit the data better than the model with one additional class. We estimated all models with a sufficient number of random starts to achieve a replicated log-likelihood (LL) value. To decide on the number of classes, we used the Bayesian Information Criterion (BIC) which should be lower when compared to the k-1 class solution. We further used the Lo–Mendell–Rubin Likelihood Ratio Test (LMR–LRT), and the Bootstrapped Likelihood Ratio Test (BLRT). These tests evaluate the adequacy of a k-1 class solution compared to a k-class solution, whereby a significant difference indicates that the k-class solution fits the data better. We did not consider solutions in which classes contained 5% of the total sample or less. Further parsimoniousness and theoretical meaning of the classes were also considered. Following class enumeration, we examined predictors associated with class membership (i.e., age, gender, and SES). We used a 3-step maximum likelihood (ML) procedure that adjusts for classification errors (Vermunt 2010), and the AUXILIARY and R3STEP commands of Mplus (Asparouhov and Muthen 2014).

Finally, to explore the associations between class membership and cognitive biases, we used the longitudinal measures of social interpretation bias, non-social interpretation bias, and memory bias to build three latent growth curve models. The factor loadings were fixed to 0 and 1 for the W1 and W3 manifest variables, respectively, and freely estimated for W2 (with similarity constraints across classes), so that the slope refers to a change between the first and third wave. We estimated multiple group models to test if the structural parameters (i.e., intercepts and slopes) differed between classes. Therefore, we compared the fit of a constrained model in which equality constraints were put on the intercepts and slopes to an unconstrained model in which the growth parameters could differ across classes. If the constrained model fit significantly worse than the unconstrained model, the growth parameters varied by class. We conducted the analyses in Mplus 7.4 (Muthén and Muthén 1998-2015) and used the estimator MLR (maximum likelihood estimation with robust standard errors) and TYPE = COMPLEX (when applicable) to account for non-normality, stratification, and non-independence of observations. Full information maximum likelihood estimation (FIML) was used to handle missing data.

## Results

Correlations between anxiety and depression at W1, W2, and W3 are presented in Appendix 1 Table 5. Please refer to Booth et al. (2019) for further descriptive statistics (e.g., mean, standard deviation) for all measures by wave.

### Overall Symptom Trajectories

**Fig. 1:**
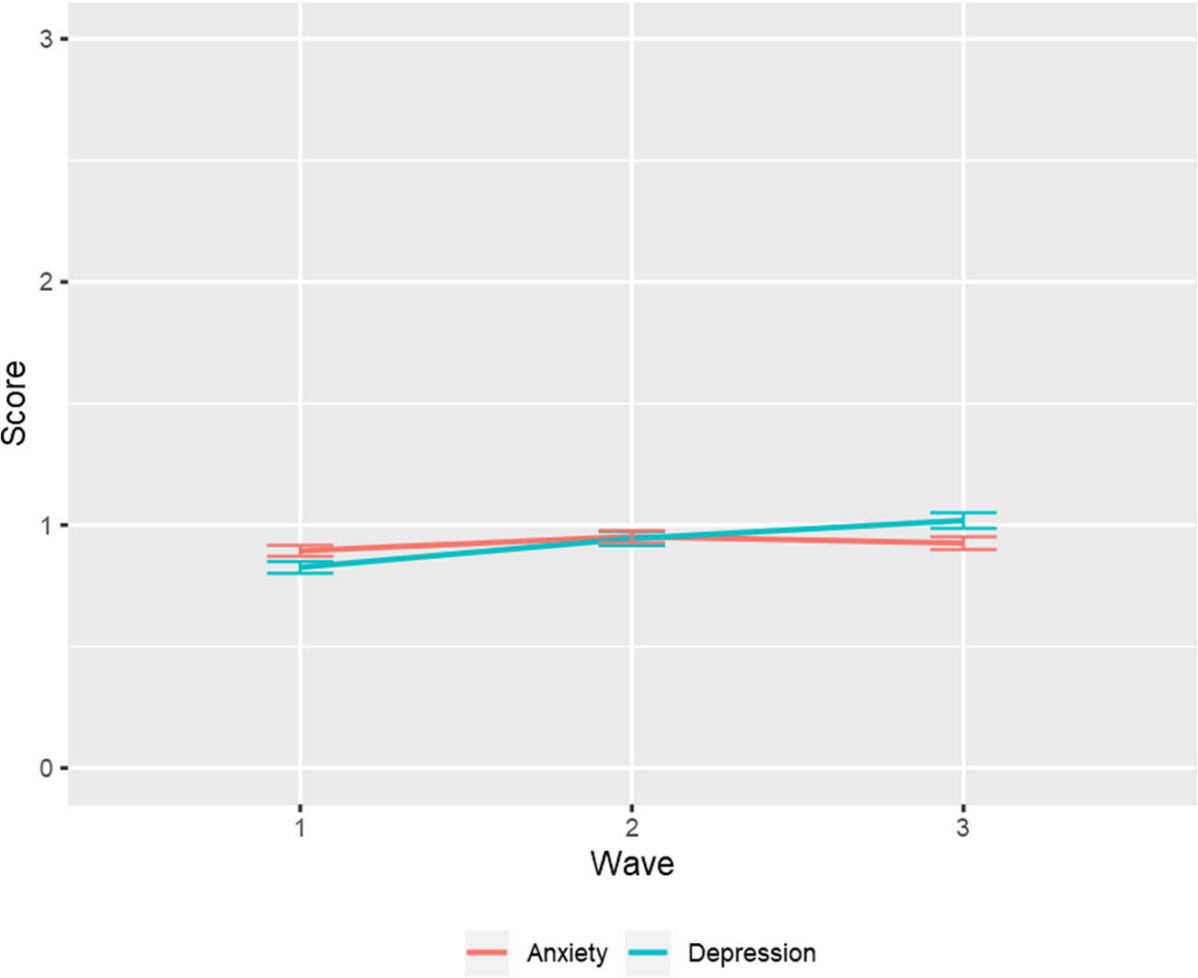
Overall levels of anxiety and depression and rate of change over time based on sample estimated means

A parallel process latent growth curve model was used to estimate overall levels of anxiety and depression and rate of change over time. The model fit the data well: χ^2^(df) = 5.29 (4), *p* = 0.529, TLI = 0.996, CFI = 0.999, RMSEA = 0.025, 90% C.I. (0.000, 0.076), SRMR = 0.023, and the results indicated that adolescents had on average low and stable levels of anxiety *b(SE)* = 0.89 (0.02), *p* < 0.001, *m(SE)* = 0.03 (0.03), *p* = 0.198, and low and increasing levels of depression *b(SE)* = 0.82 (0.02), *p* < 0.001, *m(SE)* = 0.19 (0.03), *p* < 0.001 (Fig. 1).

To examine whether anxiety and depression levels and slopes were significantly different from each other, we compared the fit of two constrained models in which they were fixed to be the same to the fit of the unconstrained model in which they could differ. If the constrained models fit significantly worse, this indicated that intercepts and slopes were different. As we used MLR, we adjusted χ^2^ using the Satorra-Bentler scaling correction.^1^ The results pointed towards similar levels (*p* = 0.886) of anxiety and depression, and a stronger increase in depression compared to anxiety, χ^2^(1) = 5.49, *p* = 0.019. To examine how anxiety and depression influenced each other over time, we looked at the associations between the intercepts and slopes. Higher initial levels of anxiety were associated with higher levels of depression (*r* = 0.21), weaker increases in anxiety (*r* = −0.12), and weaker increases in depression (*r* = −0.07). Higher initial levels of depression were associated with weaker increases in depression (*r* = −0.09), and weaker increase in anxiety (*r* = −0.11). An increase in anxiety correlated positively with an increase in depression (*r* = 0.22, all *p*s < 0.001).

### Classes of Symptom Trajectories

An unconditional, parallel process growth mixture model was specified to identify distinct developmental classes with regard to anxiety and depressive symptoms. The model fit statistics of the class solutions are presented in Table 1. BIC and LMR-LRT pointed toward a four-class solution, the BLRT toward a six-class solution, which we rejected for parsimony and because of two classes that each contained less than 5% of the sample.

**Table 1:** Model fit statistics, Growth mixture modelling analyses and class sizes

Classes	BIC	LMR–LRT	BLRT	Entropy	n_1_	n_2_	n_3_	n_4_	n_5_	n_6_
2	2735.98	−1325.13 *	−1325.13 ***	0.906	463	41				
3	2707.18	−1280.87 **	−1280.87 ***	0.839	411	51	42			
**4**	**2693.85**	**−1250.92 +**	−1250.92 ***	**0.813**	**361**	**65**	**47**	**32**		
5	2701.80	−1228.70	−1228.70 ***	0.794	329	57	51	34	33	
6	2708.32	−1217.12	1217.12 ***	0.811	320	54	54	42	22	13

Class sizes are reported based on the estimated posterior probabilities. Higher-class solutions were inadmissible. Boldface highlights the four-class solution selected based on model fit

* *p* < 0.05, ** *p* < 0.01, *** *p* < 0.001

**Fig. 2:**
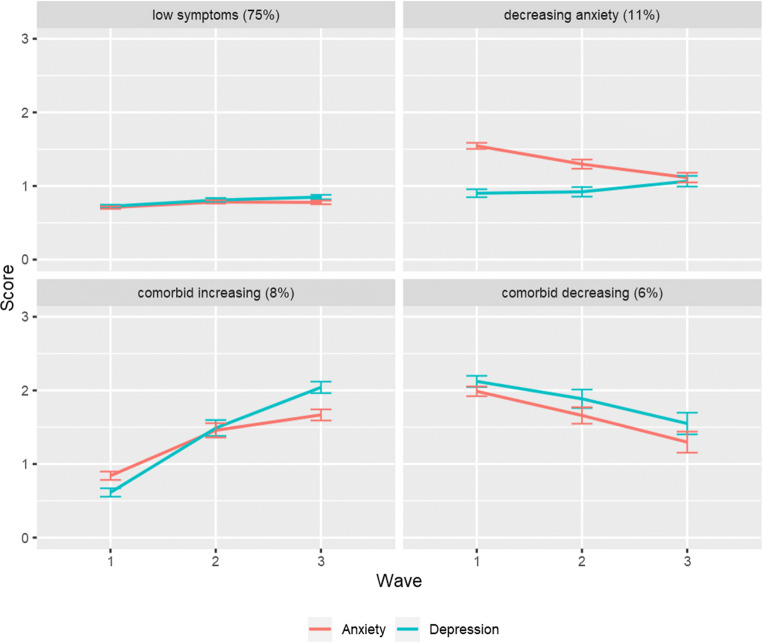
Four distinct classes of the co-development of anxiety and depression over time. Figure based on sample estimated means

Figure 2 shows the four classes with distinct co-development of anxiety and depression. Plots of all class solutions are presented in Appendix 2 Figs. 4, 5, 6, 7, and 8. Adolescents in Class 1 (75%) had consistently low levels of anxiety, and low, slightly increasing levels of depression (i.e., ‘Low symptoms’ group) [Anxiety: *b(SE)* = 0.70 (0.03), *m(SE)* = 0.07 (0.03), *p* = 0.057; Depression: *b(SE)* = 0.72 (0.03), *m(SE)* = 0.10 (0.04), *p* = 0.006]. Adolescents in Class 2 (11%) had moderate and decreasing levels of anxiety, and consistently low levels of depression (i.e., ‘Decreasing anxiety symptoms’ group) [Anxiety: *b*(*SE*) = 1.48 (0.09), *m*(*SE*) = −0.38 (0.09), *p* < 0.001; Depression: *b*(*SE*) = 0.89 (0.09), *m*(*SE*) = 0.18 (0.10), *p* = 0.091]. Adolescents in Class 3 (8%) had initially low and simultaneously increasing levels of anxiety and depression (i.e., ‘Comorbid increasing symptoms’ group) [Anxiety: *b(SE)* = 0.84 (0.07), *m(SE)* = 0.83 (0.14); Depression: *b(SE)* = 0.63 (0.07), *m(SE)* = 1.41 (0.15), *p*s < 0.001]. Adolescents in Class 4 (6%) had relatively high levels of anxiety and depression at the beginning of the study that simultaneously decreased over time (i.e., ‘Comorbid decreasing symptoms’ group) [Anxiety: *b*(*SE*) = 1.97 (0.14), *m*(*SE*) = −0.66 (0.12); Depression: *b*(*SE*) = 2.10 (0.14), *m*(*SE*) = −0.61 (0.14), *p*s < 0.001].

The four classes differed significantly in their initial level of anxiety (all *p*s < 0.008), except for the ‘Low symptoms’ and ‘Comorbid increasing symptoms’ groups, who both had relatively low levels of anxiety, Wald χ^2^(1) = 2.87, *p* = 0.090. The rate of change in anxiety also differed across the classes (*p*s < 0.001), except for a more similar decrease in the ‘Decreasing anxiety symptoms’ and ‘Comorbid decreasing symptoms’ groups, Wald χ^2^(1) = 3.00, *p* = 0.084. Initial levels of depression were similar and low in the ‘Low symptoms’ and ‘Comorbid increasing symptoms’ groups, Wald χ^2^(1) = 1.47, *p* > 0.226, and in the ‘Low symptoms’ and ‘Decreasing anxiety symptoms’ groups, Wald χ^2^(1) = 3.46, *p* = 0.063, but significantly higher in the ‘Comorbid decreasing symptoms’ group compared to the other three classes (*p*s < 0.001), and also higher in the ‘Decreasing anxiety symptoms’ group compared to the ‘Comorbid increasing symptoms’ group, Wald χ^2^(1) = 6.10, *p* = 0.014. The rate of change in depression differed across the Classes (*p*s < 0.001), except for a similar small increase in the ‘Low symptoms’ and ‘Decreasing anxiety symptoms’ groups, Wald χ^2^(1) = 0.48, *p* = 0.489.

The predictors of class membership are shown in Table 2. The results from the multinomial logistic regression using the 3-step ML procedure showed that age at the onset of the study was not related to class membership, but gender and SES were. Females were more likely to be in the ‘Decreasing anxiety symptoms’, ‘Comorbid increasing symptoms’, or ‘Comorbid decreasing symptoms’ groups than the ‘Low symptoms’ group. Participants with higher SES were more likely to be in the ‘Low symptoms’ group than the ‘Comorbid increasing symptoms’ and ‘Comorbid decreasing symptoms’ groups, and more likely to be in the ‘Decreasing anxiety symptoms’ group than the ‘Comorbid increasing symptoms’ and ‘Comorbid decreasing symptoms’ groups.

**Table 2:** Results of logistic regression analyses predicting class membership by demographic characteristics

	C3 vs C2	C1 vs C2	C4 vs C2	C1 vs C3	C4 vs C3	C1 vs C4
Variables	β (SE)	β (SE)	β (SE)	β (SE)	β (SE)	β (SE)
Age W1	−0.23 (0.20)	−0.46 (0.24)	−0.29 (0.19)	−0.24 (0.27)	−0.07 (0.24)	−0.17 (0.27)
SES	0.17 (0.17)	−0.52 (0.17) **	−0.31 (0.15) *	−0.68 (0.23) **	−0.48 (0.22) *	−0.21 (0.21)
Gender	0.97 (0.39) *	2.78 (1.00) **	2.73 (1.03) **	1.81 (1.05)	1.75 (1.13)	0.06 (1.41)

We used the auxiliary function and the R3STEP approach of MPlus to test each demographic characteristic separately. C1 = ‘Comorbid increasing symptoms’, C2 = ‘Low symptoms’, C3 = ‘Decreasing anxiety symptoms’, C4 = ‘Comorbid decreasing symptoms’. Gender: 0 = male, 1 = female. SES values ranged from 0 to 6 with 6 indicating the highest SES

* *p* < 0.05, ** *p* < 0.01

Further analyses were conducted to determine whether class membership or participant’s gender, age, SES, depression, anxiety, or cognitive biases at W1 were systematically related to whether or not they completed all three data collection waves (Gutenbrunner et al. 2018). Chi-square tests showed no significant relationship between attrition (completers vs. non-completers) and class membership (χ^2^(3) = 2.61, *p* = 0.455) or SES (χ^2^(18) = 28.13, *p* = 0.060), but there was a significant relationship between attrition and gender (χ^2^(1) = 6.47, *p* = 0.011), with more females (58%) completing all three waves compared to males (42%). A one-way MANOVA was carried out with attrition as the independent variable and age, anxiety, depression, interpretation bias, and memory bias at W1 as the dependent variables. There was a significant overall multivariate group effect for attrition (*F*(5, 486) = 2.49, *p* = 0.030, Wilk’s Λ = 0.975, η^2^ = 0.025). Follow up univariate ANOVAs indicated that there were no significant relationships between attrition and anxiety, depression, interpretation bias, and memory bias at W1 (all *ps* > 0.05). However, there was a significant relationship between attrition and age (*F*(1, 490) = 8.46, *p* = 0.004, η^2 =^ 0.017), suggesting that those who completed all three waves of data collection were slightly older at W1 (*M* = 13.42, *SD* = 0.70) compared to non-completers (*M* = 13.20, *SD* = 0.83).

### Class Membership and Cognitive Bias Trajectories

To examine the associations between class membership and cognitive biases, we used the longitudinal measures of social interpretation bias, non-social interpretation bias, and memory bias to build three latent growth curve models. For the social interpretation bias model, we fixed the residual variance of the W1 manifest variable in the ‘Comorbid increasing symptoms’ group and the residual variance of the W3 manifest variable in the ‘Comorbid decreasing symptoms’ group to zero. For the non-social interpretation bias model, we fixed the residual variance of the slopes in the ‘Comorbid increasing symptoms’ and ‘Comorbid decreasing symptoms’ group to zero, and for the memory bias model, we fixed the variance of the slope in the ‘Comorbid decreasing symptoms’ group to zero. These constraints were necessary because of negative variances, which are by definition not possible. We fixed the variances to zero because all variances were small negative values and not significant. The models fit the data well, and the model fit statistics for the unconstrained multiple group models are shown in Table 3.

**Table 3:** Model fit statistics unconstrained multiple group models

	χ^2^(df)	*p* for χ^2^	TLI	CFI	RMSEA	90% C.I.	SRMR
Social bias	5.89 (5)	.318	0.992	0.997	0.037	0.000, .134	0.030
Non-social bias	2.11 (7)	.954	1.042	1.000	0.000	0.000, 0.000	0.020
Memory bias	6.55 (5)	.256	0.982	0.993	0.050	0.000, .141	0.028

**Fig. 3:**
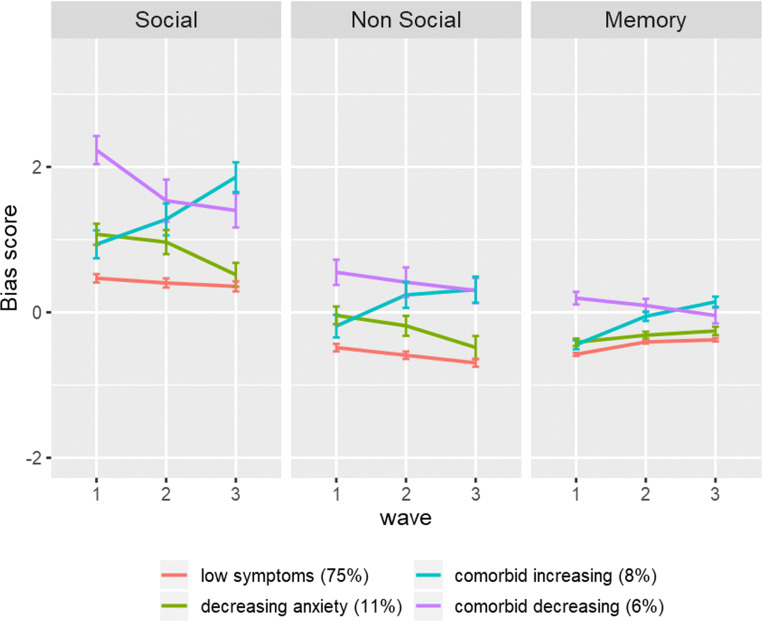
Development of cognitive biases according to class membership. Figures based on sample estimated means

**Fig. 4:**
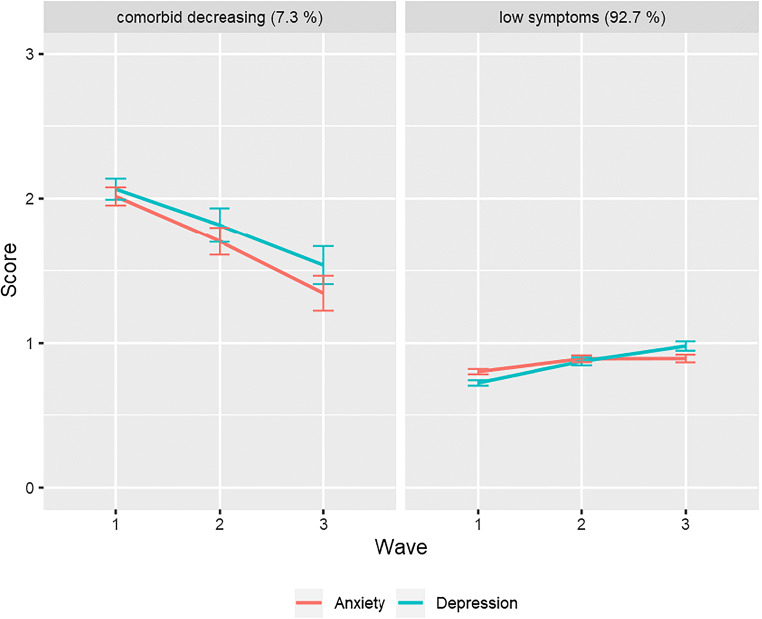
2-class solution

**Fig. 5:**
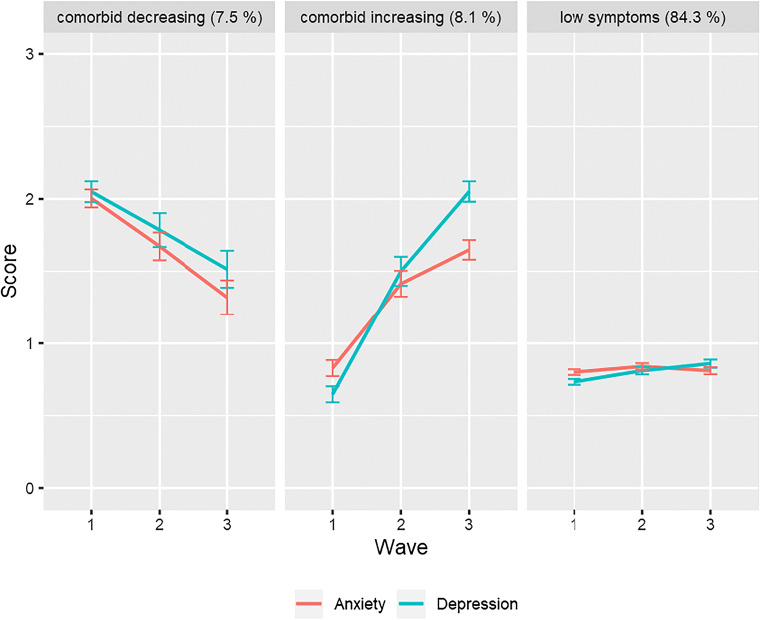
3-class solution

**Fig. 6:**
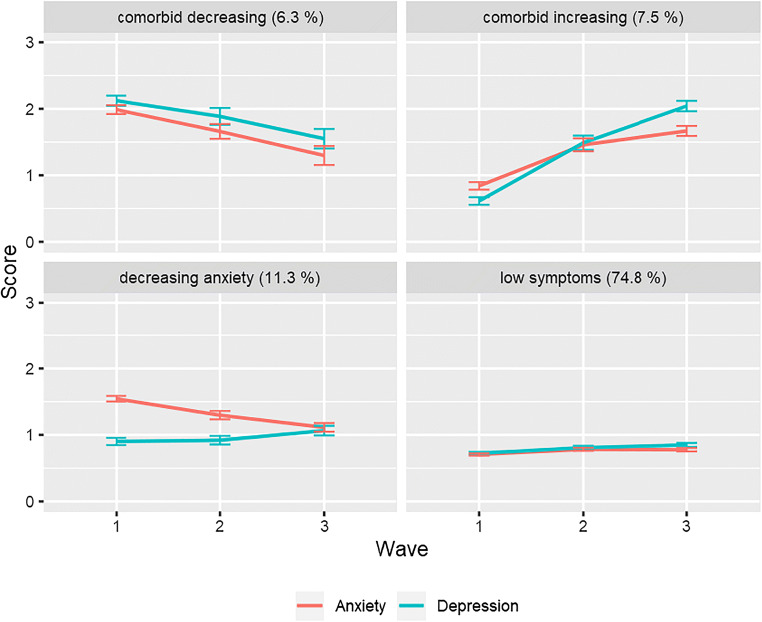
4-class solution

**Fig. 7:**
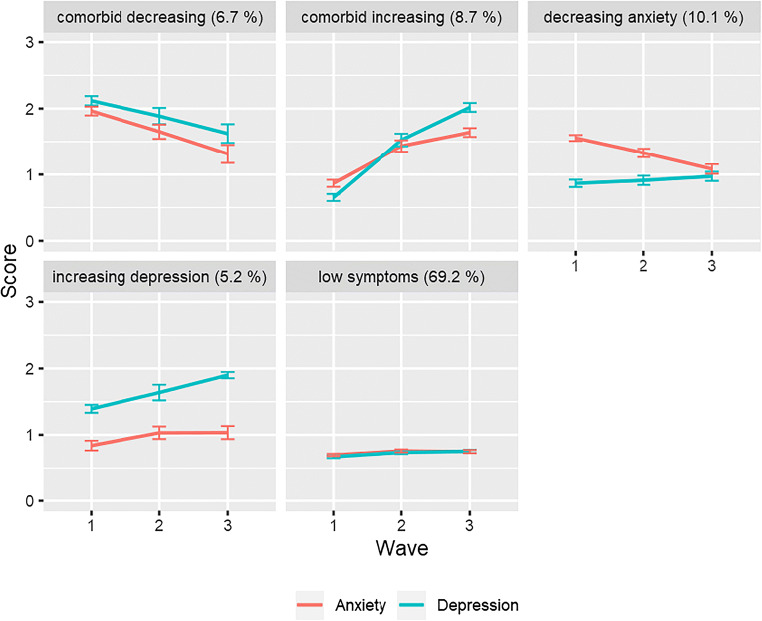
5-class solution

**Fig. 8:**
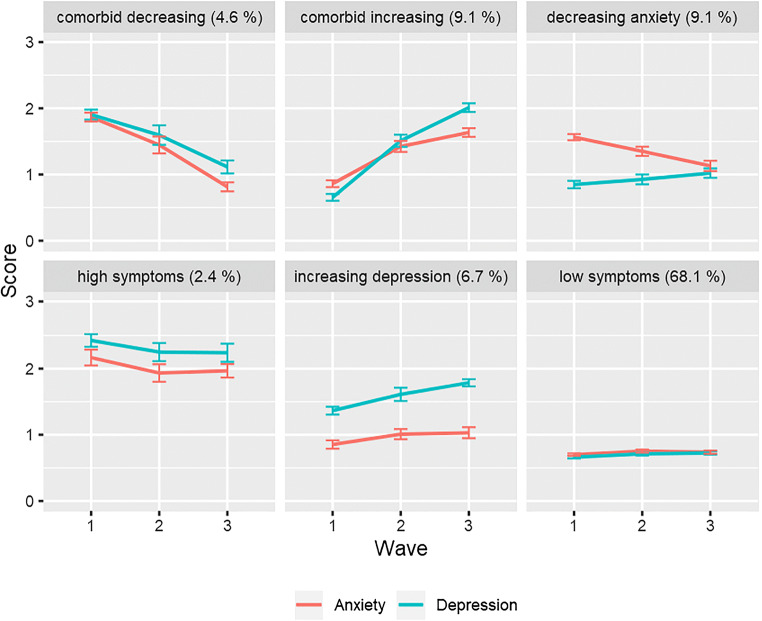
6-class solution

The results of the multiple group comparisons are shown in Table 4. In terms of class membership, the ‘Low symptoms’ group displayed a slight negative social bias at W1, which became more positive across waves. They displayed a positive non-social bias at W1, which became more positive across waves. Finally, they displayed a positive memory bias at W1, which became more negative across waves. The ‘Decreasing anxiety symptoms’ group displayed a negative social bias at W1, which became more positive across waves. They displayed a positive non-social bias at W1, which became more positive across waves. Finally, they displayed a positive memory bias at W1, which became more negative across waves. The ‘Comorbid increasing symptoms’ group displayed a negative social bias at W1, which became more negative across waves, reflecting the only increase across groups. They displayed a positive non-social bias, which became more negative across waves, again reflecting the only increase across groups. Finally, they displayed a positive memory bias at W1, which became more negative over time. The ‘Comorbid decreasing symptoms’ group displayed a very negative social bias at W1, which became more positive across waves. They displayed the only negative non-social bias at W1, which showed no change across waves. Finally, they were the only group to display a negative memory bias at W1, which became more positive across waves. These effects are visualised in Fig. 3. As a robustness check, we repeated the analysis using a manual 3-step approach that incorporates the imprecision of class assignment. The results are very similar (see Appendix 3).

**Table 4:** Results multiple group comparisons

		Low symptoms	Decreasing anxiety	Comorbid increasing	Comorbid decreasing
Social bias	Intercept	0.46 (0.06) _a_	1.10 (0.14) _b_	0.92 (0.19) _b_	2.21 (0.22) _c_
	Slope	−0.15 (0.07) * _a_	−0.59 (0.21) ** _b_	0.98 (0.23) *** _c_	−0.82 (0.22) *** _b_
Non-social bias	Intercept	−0.48 (0.05) _a_	−0.03 (0.12) _b_	−0.17 (0.16) _a,b_	0.54 (0.16) _c_
	Slope	−0.20 (0.06) ** _a_	−0.35 (0.18) * _a_	0.52 (0.18) ** _b_	−0.24 (0.13) _a_
Memory bias	Intercept	−0.58 (0.02) _a_	−0.42 (0.05) _b_	−0.45 (0.06) _b_	0.21 (0.08) _c_
	Slope	0.20 (0.02) *** _a_	0.17 (0.06) ** _a_	0.58 (0.08)*** _b_	−0.18 (0.08) * _c_

Unstandardized effects (standard errors in parentheses). Positive signs reflect negative bias scores and negative signs reflect positive bias scores. χ^2^ difference tests (*df* = 1, *p* < 0.05) were conducted for each pair of classes and adjusted using the Satorra-Bentler scaling correction. Equal sub letters in a row denotes similarity across classes

* *p* < 0.05, ** *p* < 0.01, *** *p* < 0.001

## Discussion

The present study investigated the development of anxiety and depressive symptom trajectories and cognitive biases during adolescence. As hypothesised, the results showed that overall levels of anxiety and depression were low, yet depressive symptoms increased slightly at each wave. Furthermore, in line with our hypotheses, we found multiple class trajectories of anxiety and depression. Although we did not hypothesise the number of class trajectories, our results identified four distinct developmental classes. Analysis of the development of cognitive biases with regard to class membership was in line with expectations. The majority of the sample (‘Low symptoms’ group) showed a healthy trajectory characterised by consistently low levels of anxiety, and low, but slightly increasing levels of depression. In terms of cognitive biases, the ‘Low symptoms’ group showed low interpretation and memory bias across waves. The ‘Decreasing anxiety symptoms’ group showed moderate and decreasing levels of anxiety and stable low depression. Interestingly, this group showed decreasing interpretation bias, but increasing memory bias. The ‘Comorbid increasing symptoms’ group displayed simultaneously increasing levels of anxiety and depression and similarly showed increasing interpretation and memory bias. While the ‘Comorbid decreasing symptoms’ group showed relatively high levels of anxiety and depression that simultaneously decreased over time, as well as decreasing interpretation and memory bias. These findings shed light on the different pathways of anxiety and depressive symptoms in adolescence and the co-occurring development of cognitive biases.

Consistent with previous longitudinal studies, we found substantial heterogeneity in the developmental trajectories of anxiety and depressive symptoms across adolescence (Allan et al. 2014; Cummings et al. 2014; McLaughlin and King 2015; Miers et al. 2013; Olino et al. 2010; Shore et al. 2018; Rice et al. 2019; Van Oort et al. 2009). Our findings indicate that anxiety and depressive symptoms are highly comorbid throughout this period (Hankin et al. 2016). However, there was evidence to suggest that anxiety and depression trajectories develop as distinct parallel growth processes, which supported previous research (Cummings et al. 2014; Hale III et al. 2009). In addition, we found that the development of interpretation and memory biases, matched the class trajectories of anxiety and depression. These findings provide insight into the potential risk and protective factors that may contribute to levels of anxiety and depressive symptoms in adolescence. The four distinct class trajectories identified and their associated cognitive biases are discussed below.

The ‘Low symptoms’ group displayed consistently low levels of anxiety and depression, with a slight increase in depression over time. This group are perhaps representative of the non-clinical proportion of adolescents assessed in the study, indicating a healthy pathway of development for adolescents with low-risk of developing psychopathology. Given the age range of our sample, this pattern is consistent with evidence in the literature, which suggests that the onset of anxiety often occurs during childhood, while depression tends to develop during adolescence (Hankin et al. 1998; Merikangas et al. 2010; Roza et al. 2003). The ‘Low symptoms’ group displayed a small decrease in interpretation bias and a small increase in memory bias over time. The increase in negative memory bias is perhaps linked to the slight increase in depressive symptoms, suggesting that negative memory bias may be more closely associated with depression than anxiety. This is in line with previous research, which shows that depressive symptoms are associated with enhanced recall for negative compared to positive self-referent information (Lau and Waters 2016; Platt et al. 2016). However, compared to the other groups, adolescents with ‘Low symptoms’ showed lower levels of negative interpretation bias and negative memory bias, suggesting that their bias towards positive, as opposed to negative processing, may act as protective mechanisms against the development of anxiety and depression.

Twenty-five percent of the sample showed elevated symptoms of anxiety and depression at some point, which supports previous epidemiological evidence (NHS Digital, 2017). The ‘Decreasing anxiety symptoms’ group, showed moderate but decreasing levels of anxiety and stable low depressive symptoms. This developmental trajectory indicates that anxiety was more severe during early adolescence, but as anxiety levels decreased over time, symptoms became level with depression. However, there was a non-significant increase in depressive symptoms in this group, which matched the magnitude of increase in the ‘Low symptoms’ group. This could be attributed to low power, due to the small number of participants in the ‘Decreasing anxiety symptoms’ group. This trajectory is consistent with Pathway one outlined in the Multiple Pathways Model (Cummings et al. 2014), which describes youth that have a predisposition for anxiety that later becomes comorbid with depressive symptoms. Furthermore, adolescents in the ‘Decreasing anxiety symptoms’ group showed a decrease in interpretation bias, which suggests that less negative (or more positive) interpretations of ambiguous scenarios are protective factors associated with decreasing anxiety levels. This is consistent with a recent meta-analysis that showed a robust association between high anxiety and negative interpretation bias in children and adolescents (Stuijfzand et al. 2017). In contrast, negative memory bias increased over time in the ‘Decreasing anxiety symptoms’ group, which was a consistent finding across the ‘Low symptoms’ and ‘Comorbid increasing symptoms’ groups, suggesting that this is an adolescent-typical effect.

The ‘Comorbid increasing symptoms’ and ‘Comorbid decreasing symptoms’ groups provide support for Pathway two of the Multiple Pathways Model, where anxiety and depressive symptoms co-develop simultaneously. Cognitive biases may reflect transdiagnostic risk factors in this pathway (Cummings et al. 2014). In our sample, the ‘Comorbid increasing symptoms’ group showed increasing levels of anxiety and depression over time, as well as increasing negative interpretation and negative memory biases. Whereas the ‘Comorbid decreasing symptoms’ group, showed initially high but decreasing levels of anxiety and depression, and decreasing negative interpretation and negative memory biases over time. Therefore, the cognitive bias pathways matched the direction of symptoms, indicating that biases are key risk factors associated with anxiety and depressive symptoms. Overall, these two groups showed the most elevated symptoms of anxiety and depression and displayed more negative interpretation and memory bias, compared to the other groups. This suggests that anxiety and depression share a high degree of overlap and common risk and protective mechanisms that may worsen or improve overall adjustment in adolescence. Thus, our study provided evidence for Pathway one and two outlined in the Multiple Pathways Model. We found no evidence for Pathway three, which is characterised by a diathesis for depression and later comorbid anxiety. However, we did not expect to observe evidence for Pathway three, which is thought to distinguish older adolescents and adults.

The development of cognitive biases in relation to anxiety and depression is a novel aspect of the present study. We found that the development of interpretation and memory biases corresponded to the anxiety and depressive symptom trajectories in the four distinct groups, suggesting that they are closely related. Increasing negative memory bias over time was present in all groups, apart from the ‘Comorbid decreasing symptoms’ group, and therefore may reflect a normative effect. Adolescence is an important phase of developing a sense of identity and studies have shown that negative self-evaluations are highly prevalent amongst depressed adolescents (Orchard et al. 2019). Thus, as adolescents get older they may become more sensitive to negative self-perceptions and recalling negative thoughts. Furthermore, all groups showed decreasing social and non-social interpretation bias over time, except for the ‘Comorbid increasing symptoms’ group. Together, this pattern suggests that negative interpretation bias is more prominent in younger adolescence, while negative memory bias is more characteristic of mid-adolescence.

Social bias was predominantly more negative than any other bias, indicating that negative interpretations of social scenarios were relatively high across all groups. This is perhaps related to adolescence in general, as this developmental period is characterised by significant neurodevelopmental changes and heightened sensitivity, in particular to the social environment and peers (Fuhrmann et al. 2015; Nelson et al. 2016). Heightened levels of social bias in our sample are therefore likely to reflect changes in information-processing, as adolescents become more sensitive to social input from peers and are more vulnerable to negative social situations. Negative social bias was highest in the ‘Comorbid decreasing symptoms’ group at W1 and this decreased over time. In the ‘Comorbid increasing symptoms’ group, social bias became more negative across waves, reflecting the only increase across groups. Furthermore, in this at-risk group, social bias was markedly higher than non-social bias and memory bias across all three waves. This pattern indicates that negative social biases may play a role in increasing comorbid anxiety and depression symptom trajectories and are perhaps potential targets for early interventions.

One challenge for early intervention approaches is being able to identify adolescents who are the most at-risk (e.g., the ‘Comorbid increasing symptoms’ group) from those who may show a natural decrease in symptom trajectories over time (e.g., the ‘Comorbid decreasing symptoms’ group). It is important to note that because we only assessed three time points we cannot say with any certainty that those in the ‘Comorbid decreasing symptoms’ group would continue to show a decrease in symptoms. Previous research using more time points, suggests that some at-risk adolescents display high and fluctuating symptoms (Shore et al. 2018). Therefore, it is possible that this seemingly improving group would show a spike in symptoms at later stages of adolescence, particularly as their symptoms and negative biases remained at a similar level to the ‘Comorbid increasing symptoms’ group. Further investigation of other factors such as SES, gender, age, school type, peers, friendships, social support, family environment, or genetics, in addition to cognitive biases, may help to identify adolescents who are at greatest risk of comorbid increasing anxiety and depression trajectories (Field and Lester 2010; Van Harmelen et al. 2017), and thus benefit the most from early interventions targeting cognitive biases.

We found evidence in our sample that being female and having lower SES were risk factors for elevated symptoms of anxiety and depression, which is consistent with previous research (Shore et al. 2018; Van Oort et al. 2009). Females were more likely to be in the ‘Decreasing anxiety symptoms’, ‘Comorbid increasing symptoms’, or ‘Comorbid decreasing symptoms’ groups than the ‘Low symptoms’ group. Furthermore, adolescents with higher SES were more likely to be in the ‘Low symptoms’ and ‘Decreasing anxiety symptoms’ groups, suggesting lower risk to psychopathology over time, perhaps due to greater access to resources or certain environmental or familial advantages. The ‘Comorbid increasing symptoms’ group showed the lowest level of SES, therefore lack of resources or familial disadvantage may have contributed to this poor trajectory. This may also reflect a gender interaction, as previous research has found that the greatest negative impact of low SES was found in older adolescent girls (Patalay and Fitzsimons 2018). This highlights the importance of taking into consideration demographic characteristics as well as social, biological, or cognitive factors that might influence risk and resilience for psychopathology, which may be important for identifying adolescents and developing more targeted interventions.

Given that symptoms of anxiety and depression often persist beyond childhood, through adolescence and into adulthood, prevention and early intervention programmes are key. The findings of the present study identify potential cognitive biases that may be useful to target in the development of anxiety and depressive symptoms in early to mid-adolescence. However, these results should be interpreted with caution and future research directly targeting these cognitive mechanisms in intervention studies and randomized controlled trials in adolescents is needed, particularly in clinical and at-risk youths. Further, as our sample included healthy adolescents with elevated symptoms, rather than a clinical sample, we can only make inferences about the early development of anxious and depressive symptoms and the suggestion that cognitive biases may exacerbate psychopathology (Fox and Beevers 2016). Nonetheless, this study has the potential to inform future research on the trajectories of anxiety and depressive symptoms in early to mid-adolescence in a normative sample and associations with the development of interpretation and memory biases.

The present study has a number of strengths. First, we used a longitudinal design to assess adolescent developmental psychopathology across multiple time points, within a large normative sample. This provides valuable insights into the symptom trajectories of anxiety and depression in healthy adolescents and the development of psychopathology in the early stages. Second, to the best of our knowledge, this study was the first to investigate cognitive biases longitudinally. This novel aspect of the study sheds light on the development of interpretation bias and memory bias during adolescence and highlights associations between cognitive biases and trajectories of anxiety and depressive symptoms. Third, we used a person-oriented approach (i.e., GMM), which allowed us to identify distinct subgroups of adolescents with varying levels and rates of change in anxiety and depression. Finally, we were able to retain a large sample across three waves and used a wide range of behavioural and self-report measures. Therefore, the variability within our sample allowed us to investigate developmental trajectories of anxiety and depression, and advance current knowledge of the cognitive factors associated with adolescent psychopathology.

However, there are several limitations to the study worth noting. One limitation is that there may be other biological, social, or cognitive factors (e.g., genetics, temperament, executive functions, peers, friendships, social support, family environment or parental behaviours) associated with developmental trajectories of anxiety and depression in adolescence (Field and Lester 2010; Van Harmelen et al. 2017). In the present study, we focused on cognitive biases, based on previous literature that highlights the importance of attention, interpretation, and memory biases in youth (Lau and Waters 2016). We were unable to include attention bias, which may be important, in our analysis due to low internal consistency and poor psychometric properties of the Dot-probe task (Booth et al. 2019). It is important for future research to examine multiple cognitive biases, such as the development of attention, interpretation, and memory bias longitudinally, in order to assess cognitive models that emphasise the importance of these information-processing biases in anxiety and depression in greater detail.

Another limitation is that we did not examine the trajectories of specific anxiety disorder symptoms (i.e., generalised anxiety, social anxiety, separation anxiety etc.), which may have influenced the results. Less research has examined the developmental trajectory of anxiety, yet it is likely that different anxiety subtypes show different developmental pathways (Cummings et al. 2014; Hale III et al. 2009; Van Oort et al. 2009). However, we were unable to assess anxiety sub-types, due to not enough power to investigate numerous models. In addition, we used the short version of the RCADS questionnaire, which is designed primarily to assess anxiety symptoms as a whole. Future studies in adolescence could test larger samples and use the long version of the RCADS to disentangle the trajectories of anxiety sub-types further.

Finally, growth mixture modeling has received some criticism in the literature (Petersen et al. 2019). One criticism to this approach is that growth mixture models are essentially clustering procedures yielding sample specific results. However, the class trajectories identified in our sample are in line with previous studies that have investigated the developmental pathways of anxiety and depression in adolescents (Cummings et al. 2014), supporting the validity of our findings. More large-scale longitudinal studies in normative samples are needed to replicate our findings. Another problem with this approach is that growth mixture models are not the preferred method to study temporal relations. Whilst these relations were not the primary focus of the current study, prior work suggests that cognitive biases play a key role in the development and maintenance of internalising disorders (Lau and Waters 2016; Mathews and MacLeod 1994; Muris and Field 2008). However, these relationships have not been investigated in adolescents longitudinally, which would be an important direction for future research. In addition, further research using analytical approaches such as cross-lagged panel models or experimental designs such as Cognitive Bias Modification (CBM) studies or randomized controlled trials that target interpretation or memory biases in adolescents would provide a deeper insight into temporal relations and directionality (Lau and Pile 2015). Finally, future longitudinal research should investigate the developmental period of younger children or later adolescence to provide a more holistic picture of when cognitive biases are likely to develop and how they are associated with anxiety and depression over the lifespan (Field and Lester 2010).

In summary, this study investigated the development of anxiety and depressive symptom trajectories in adolescence and the co-occurring development of cognitive biases. We found evidence for four distinct developmental classes of anxiety and depression and demonstrated that interpretation and memory biases are risk and protective factors associated with symptom trajectories. This novel study sheds light on the longitudinal development of social interpretation bias, non-social interpretation bias, and memory bias across adolescence. Negative social interpretation bias was particularly high in our sample, which may reflect high sensitivity to peers or the social environment, behaviours typically observed in this age group. Additional longitudinal research investigating cognitive biases and further replication of this approach with larger samples is required to validate the class trajectories identified in our sample. The current study advances our understanding of the developmental trajectories of psychopathology in early to mid-adolescence and has the potential to inform future research on potential cognitive mechanisms to target for prevention and early interventions.

## Appendix 1

### Correlations

**Table 5:** Correlations for anxiety and depression at each wave

Measures	1	2	3	4	5
1. Anxiety W1	_				
2. Anxiety W2	0.64**	_			
3. Anxiety W3	0.49**	0.67**	_		
4. Depression W1	0.75**	0.50**	0.37**	_	
5. Depression W2	0.53**	0.72**	0.50**	0.62**	_
6. Depression W3	0.42**	0.57**	0.71**	0.50**	0.71**

**p* < 0.05, ** *p* < 0.01, *** *p* < 0.001

## Appendix 2

### Appendix Class Enumeration

Appendix 2 shows plots of anxiety and depression of the 2 to 6 class solutions. It appears that the ‘Low symptoms’ and ‘Comorbid decreasing symptoms’ groups are found in all class solutions. The ‘Comorbid increasing symptoms’ group (a subdivision of the ‘Low symptoms’ group) emerges in the 3-class solution and remains to be present in higher-class solutions. The ‘Decreasing anxiety symptoms’ group (another subdivision of the ‘Low symptoms’ group) emerges in the 4-class solution and remains to be present in higher-class models. In the 5-class solution a group with ‘Increasing depression symptoms’ (a subdivision of the ‘Low symptoms’ group) emerges which appears again in the 6-class solution. Finally, there is a group with ‘High symptoms’ (a subdivision of the ‘Comorbid decreasing symptoms’ group) in the 6-class solution. Despite the potential interest in the classes that emerge in the 5 and 6-class models, the model fit statistics clearly point towards a 4-class solution.

## Appendix 3

### Manual 3-Step Approach

We also used a manual 3-step approach for the cognitive bias growth parameters to incorporate the imprecision of class membership (Asparouhov and Muthen 2014). In Step 1, we estimated the latent class model and saved the most likely class variable. In Step 2, we determined the measurement error for the most likely class variable. In Step 3, we estimated our auxiliary models. In these models, latent class membership is measured by the most likely class variable and the measurement errors are fixed based on the values computed in Step 2. Our auxiliary models were latent growth curve models. To build these, we used the longitudinal measures of social interpretation bias, non-social interpretation bias, and memory bias. The results of the manual 3-step approach (Table 6) closely resemble the results from the multiple group models in which classification errors were not taken into account.

**Table 6:** Results manual 3-step approach

		Low symptoms	Decreasing anxiety	Comorbid increasing	Comorbid decreasing
Social bias	Intercept	0.39 (0.07)	1.17 (0.18)	1.04 (0.23)	2.45 (0.18)
	Slope	−0.15 (0.08) ^+^	−0.69 (0.26) **	1.07 (0.24) ***	−0.90 (0.24) ***
Non-social bias	Intercept	−0.52 (0.06)	−0.02 (0.16)	−0.12 (0.19)	0.67 (0.22)
	Slope	−0.20 (0.07) **	−0.50 (0.20) *	0.62 (0.23) **	−0.13 (0.25)
Memory bias	Intercept	−0.62 (0.03)	−0.40 (0.08)	−0.47 (0.07)	0.43 (0.06)
	Slope	0.17 (0.03) ***	0.10 (0.07)	0.75 (0.08) ***	−0.29 (0.09) **

Unstandardized effects (standard errors in parentheses). Positive signs reflect negative bias scores and negative signs reflect positive bias scores

^+^*p* < 0.10, * *p* < 0.05, ** *p* < 0.01, *** *p* < 0.001

## Footnotes

1 When conducting χ2 difference tests using the MLR estimator in Mplus it is necessary to adjust the χ2 using the Satorra-Bentler scaling correction. More information can be found here: http://www.statmodel.com/chidiff.shtml
